# Does the Medical Student Performance Evaluation Change the Decision to Invite Residency Applicants?

**DOI:** 10.5811/westjem.2021.4.52374

**Published:** 2021-08-21

**Authors:** Terra N. Thimm, Christopher S. Kiefer, Mara S. Aloi, Moira Davenport, Jared Kilpatrick, Jeffrey S. Bush, Lindsey Jennings, Stephen M. Davis, Kimberly Quedado, Erica B. Shaver

**Affiliations:** * West Virginia University School of Medicine, Department of Emergency Medicine, Morgantown, West Virginia; † Allegheny General Hospital, Department of Emergency Medicine, Pittsburgh, Pennsylvania; ‡ Medical University of South Carolina, Department of Emergency Medicine, Charleston, South Carolina; § West Virginia University School of Public Health, Department of Health Policy, Management, and Leadership, Morgantown, West Virginia

## Abstract

**Introduction:**

Although emergency medicine (EM) residency program directors (PD) have multiple sources to evaluate each applicant, some programs await the release of the medical student performance evaluation (MSPE) to extend interview offers. While prior studies have demonstrated that MSPE content is variable and selectively positive, no prior work has evaluated the impact of the MSPE on the likelihood to invite (LTI) applicants for a residency interview. This study aimed to evaluate how information in the MSPE impacted LTI, with the hypothesis that changes in LTI would be relatively rare based on MSPE review alone.

**Methods:**

We conducted a prospective, observational study analyzing applications to three EM residency programs during the 2019–2020 match cycle. Reviewers assessed applications and rated the LTI on a five-point Likert scale where LTI was defined as follows: 1 = definitely no; 2 = probably no; 3 = unsure; 4 = probably yes; and 5 = definitely yes. The LTI was recorded before and after MSPE review. A change in LTI was considered *meaningful* when it changed the overall trajectory of the applicant’s likelihood to receive an invitation to interview.

**Results:**

We reviewed a total of 877 applications with the LTI changing ≥1 point on the Likert scale 160 (18.2%) times. The LTI was *meaningfully* impacted in a minority of applications – 48 total (5.5 %, p< 0.01) – with only 1 (0.11%) application changing from 1 or 2 (definitely/probably no) to 4 or 5 (probably/definitely yes) and 34 (3.8%) changing from 3 (unsure) to 4 or 5 (probably/definitely yes). Thirteen (1.5%) applications changed from 4 or 5 (probably/definitely yes) to 3 (unsure or probably/definitely no).

**Conclusion:**

Review of the MSPE resulted in a *meaningful* change in LTI in only 5.5% of applications. Given the time required for program leadership to review all parts of the variably formatted MSPEs, this finding supports a more efficient application review, where the PD’s focus is on succinct and objective aspects of the application, such as the Standardized Letter of Evaluation.

## INTRODUCTION

Emergency medicine (EM) program directors (PD) have multiple data points to review when screening applicants and extending interview offers. These data points include the curriculum vitae (CV), medical school transcript, United States Medical Licensing Examination (USMLE) results, personal statement, Standardized Letters of Evaluation (SLOE), and the medical student performance evaluation (MSPE). The MSPE is designed to be a letter of evaluation that provides an objective summary of a medical student’s personal attributes, experiences, and academic accomplishments, as well as a comparison to their institutional peers.^1^ The guidelines for writing the MSPE provided by the American Association of Medical Colleges (AAMC) illustrate that it should contain six sections: (1) identifying information; (2) noteworthy characteristics; (3) academic history; (4) academic progress; (5) summary; and (6) medical school information.^1^ Despite the intended purpose of the MSPE, previous literature has demonstrated that not all institutions follow the AAMC guidelines regarding letter construction.^2^,^3^

Given the average of 101 hours per year spent on application review by PDs, they desire objective and comparative data to differentiate between applicants as efficiently as possible.^4^ In EM, 83% of PDs cite the MSPE as one of many factors used to decide which applicant to invite.^5^ The potential value of the MSPE lies in the fact that it is the only place in the application where a PD can view narrative information outlining a student’s performance in both the pre-clinical and clinical curriculums, personal and professional attributes, and performance compared to peers at their institution.^1^ Unfortunately, in addition to the variability in the structure of the MSPE between institutions, prior work has demonstrated that MSPE content is selectively laudatory.^6^ The variability and overall positive tone may have contributed to prior survey data showing that EM PDs ranked the MSPE as 13^th^ of the 16 most important application components with regard to resident selection.^7^ Although this survey was done prior to the most recent MSPE taskforce recommendations instituted in 2016, the most recent National Resident Matching Program survey of PDs in EM in 2018 continued to show that specialty letters of recommendation (i.e., the SLOE) are prioritized over the MSPE in selecting applicants for interview, with the SLOE ranked as the first most influential factor out of 33 total factors surveyed and tthe MSPE ranked 23rd out of those 33 factors.^5^

Prior literature regarding the MSPE has largely focused upon the summary section, which typically includes a summative adjective or statement regarding the overall performance of the medical student. Authors of MSPEs are advised that the adjective or statement should be included only if school-wide comparative data is available.^1^ Hom et al revealed limitations in availability of comparative data with regard to the summary adjective and demonstrated that 17% of institutions using a summary adjective did not provide a full list of potential adjective words or distribution data, and an additional 10% did not provide the distribution data for each adjective.^8^ In addition, this adjective tends to be universally positive with descriptors such as “outstanding,” “excellent,” “very good,” and “good” representing the most common categories.^3^ Program directors attempting to compare students on the basis of the summary adjectives face the challenge of incomplete comparative information, inconsistent terminology between institutions, and the usage of only positive adjectives to describe performance.^9^,^10^

**Population Health Research Capsule**

What do we already know about this issue?

*The medical student performance evaluation (MSPE) is known to be selectively laudatory and variable in content. Emergency medicine (EM) program directors value objective, concise information when reviewing applicants for residency*.

What was the research question?

*Does review of the MSPE provide information that results in meaningful change in a program’s likelihood to invite (LTI) an applicant for an EM interview?*

What was the major finding of the study?

*The MSPE results in meaningful change in LTI in approximately ~5% of application reviews*.

How does this improve population health?

*Our findings support Program Directors’ focus on succinct and objective aspects of the application rather than the MSPE, such as the Standardized Letter of Evaluation*.

Given these challenges, it is not surprising that EM PDs value more succinct and objective parts of the application, such as the SLOE, clerkship grades, and EM rotation performance, when deciding which students to interview.^7^,^11^ Despite the limitations of the MSPE outlined above, some programs wait two weeks after the Electronic Residency Application Service (ERAS) opens on September 15 for the traditional release of the MSPE on October 1 before beginning comprehensive application review. This leads to a compressed time frame for completion of application review and interview offers. In this study we aimed to evaluate whether information gained from review of the MSPE changed PDs likelihood to invite (LTI) applicants for interview.^12^ Our hypothesis was that MSPE review would not consistently result in *meaningful* change in the LTI.

## METHODS

Three Accreditation Council of Graduate Medical Education-accredited EM residency programs (sites) participated in this prospective, observational study conducted during the 2019–2020 application cycle, with data collection completed between October 1, 2019–November 1, 2019. Two of the sites were university-affiliated, and one site was university-affiliated and community-based. Reviewers from each of the three participating sites reviewed applications submitted through ERAS. The application reviewers, including three PDs, three associate/assistant PDs, and one chief resident, all made final decisions regarding applicant interview invitations in the 2019–2020 cycle. The chief resident who reviewed at one study site reviewed 19 total applications, and his decisions on inviting were re-reviewed by the site PD previous to making it final, thus ensuring that the review of applications remained consistent with other applications reviewed at this site. Table 1 provides further information related to site/program demographics, class size, and total numbers of applications received and reviewed, as well as the site reviewers and associated years of experience.

Inclusion criteria for the study were EM applications received via ERAS and reviewed by the three participating residency programs. Exclusion criteria included applicants already invited for interview prior to MSPE release, applications missing an MSPE, and applications that were inadvertently reviewed by more than one reviewer at a single site. We excluded applicants who had been offered an interview prior to MSPE review, as the investigators felt that the impact of the information contained in the MSPE upon LTI could not be accurately assessed if the decision to invite had previously been made.

We acknowledge that each site has a unique approach to application review and the decision to invite is individual and multifactorial. Given that the specific objective of the study was to determine the impact of the MSPE on LTI, each site was permitted to review applications via their standard processes, reviewing all other variables as they normally would, except for being blinded to the MSPE on the initial review. Blinding was accomplished by instructing site reviewers to *not* view the MSPE in ERAS on initial application review. After this initial review, reviewers recorded their initial LTI on the Likert scale, described in the following paragraph. Subsequently, the MSPE was reviewed and the LTI was re-recorded.

The pre- and post-MSPE review LTI was determined on a five-point Likert scale: 1= definitely no; 2 = probably no; 3 = unsure; 4 = probably yes; and 5 = definitely yes. The “unsure” designation was intended for candidates placed on each program’s waitlist or those applications that the program was planning to review an additional time prior to making a final interview decision. The LTI and factors influencing the LTI on initial review were recorded on an internally derived survey developed through a secure Qualtrics platform (Qualtrics_R_^XM^, Provo, UT) (Appendix 1). All reviewers worked collaboratively to develop and test the survey before official implementation to ensure it efficiently captured relevant data that outlined the application factors influencing the applicant’s LTI both before and after MSPE review. Through a conference call with all sites prior to the initiation of the review process, all reviewers received a brief tutorial of the process for survey completion. Daily email reminders were sent to all reviewers during the study period.

Given that the same applicant could have applied to more than one of the institutions reviewing applications, each review counted as an individual data point in the study. The inclusion of multiple data points for a single applicant, derived from different review sites, was felt to be appropriate given that every program has its own system for application review and may differ in the factors that are most influential in deciding on the LTI for an applicant.

When the LTI *did* change after MSPE review, the reviewer recorded what information obtained from the MSPE resulted in the change. Options presented to reviewers for information obtained from the MSPE included the following: narrative rotation comments; class rank; report of remediation/probation; delay in completion of training; perception of professionalism; and a free-text box for other factors that influenced the LTI. Alternatively, when the LTI *did not* change after MSPE review, reviewers noted the primary source of data from the ERAS application that influenced their initial LTI. Potential data points for selection included the SLOE global assessment rankings, personal statement, prior knowledge of the applicant (i.e., had rotated at the institution, was known from medical school, etc.), CV information, USMLE performance, and another free-text box for any additional influencing factors.

Although all changes were recorded and analyzed, we only considered a change in LTI to be *meaningful* when it changed an applicant’s invitation status. For example, a change was considered *meaningful* when an interview offer was planned on initial application review (definitely yes/probably yes), but after MSPE review, the candidate’s LTI was changed to a Likert scale anchor signifying the applicant would no longer likely be invited (unsure/probably no/definitely no). Conversely, a change was considered *non-meaningful* when the change in LTI did not change the overall outcome of the applicant’s interview status. Specific examples of *non-meaningful* change in our study are demonstrated by a change from “probably yes” to “definitely yes” or “probably no” to “definitely no” that did not result in any change in the program’s LTI. Changes involving the LTI of “unsure” were considered *meaningful* when it resulted in a change in the applicant’s interview status. For example, “unsure” to “probably yes” or “definitely yes” resulted in a likely interview offer where one had not been previously planned/extended and was considered *meaningful*. A change from “unsure” to “probably no” or “definitely no” was not considered *meaningful, as the applicant had never actually received an invite, and this didn’t change with the change in the LTI from an “unsure” to a “probably or definitely no.”* To ensure that our definition of *meaningful* change was valid, we analyzed and recorded the real-world interview status of each applicant (interview offered or not offered) and compared it to the post-MSPE review “final” LTI to ensure that all applicants with a “probably yes”/“definitely yes” were invited and all applicants with an “unsure”/“probably no”/“definitely no” were not invited.

Data were extracted from Qualtrics and analyzed calculating for all variables. We assessed substantial differences in average LTI rankings between reviews that resulted in a *meaningful* LTI change vs *non-meaningful* change using analysis of variance or the nonparametric Wilcoxon test in the case of significant departures from normality. An alpha of 0.05 was selected as the threshold for statistical significance. The institutional review board at the main study site reviewed and approved this study.

## RESULTS

The three institutions received a total of 2905 applications, with 1191, 1071, and 643 applications at each site, respectively (Table 1). Following each institution’s application of their individual screening process, there were a total of 1001 applications reviewed from the three institutions during the study period.

**Figure 1:**
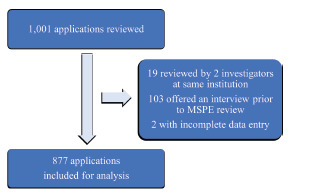
Flow of application review and analysis. *MSPE*, Medical Student Performance Evaluation.

Overall, 124 applications were excluded from review. Of these 124, 103 were offered an interview prior to MSPE review, and 19 were excluded due to inadvertent review by two investigators at the same institution. Two additional applications were excluded due to incomplete data entry. The remaining 877 applications – 244 from Site 1, 290 from Site 2, and 343 from Site 3 – were analyzed (Figure 1).

The 877 applications reviewed were from 757 unique applicants, and the demographic characteristics of the unique applicants and the study sites are shown in Table 2. Residency programs received applications from medical schools across the country, with all regions being fairly equally represented. Although a slightly larger number of applicants are reported from the study site regions of the northeast and southeast, all regions of the country are represented in the data set. For further details regarding more specifics of applicant geographic demographics, please refer to appendix B.

To determine whether the Likert scale described in the methods section correlated with the actual invite decision from programs, we analyzed the “real-world” final interview decision for each LTI rating, as displayed in Table 3. The LTI recorded in the survey instrument strongly correlated with the final interview decision by the program.

In 160 (18.2%) of the total applications, pre/post LTI changed ≥1 point on the Likert scale, but in 91 of those applications (56.8%), the overall LTI was *not meaningfully* changed, as referenced in the criteria for *meaningful*, as defined above. Therefore, in 829 (94.5%) of the total applications, there was *no meaningful* change in LTI following MSPE review (*P* = <0.001).

**Figure 2:**
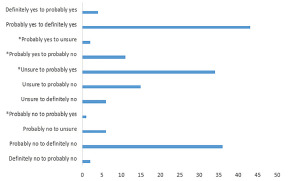
The degrees of change in “likelihood to invite” before and after Medical Student Performance Evaluation (MSPE) review for applications where MSPE review resulted in a change of at least 1 point on the Likert scale. *Indicates *meaningful* change in the likelihood to invite, defined by a change in the Likert scale from no (≤ 2) to yes (≥ 4); from yes (≥ 4) to no (≤ 2); from unsure (3) to yes (≥ 4); and from yes (≥ 4) to unsure (3). Those applicants who received a score of no (≤ 2) to unsure (3) or unsure (3) to no (≤ 2) never had a direct interview invitation offered in the course of the study, and thus this change was not considered *meaningful*. *LTI*, likelihood to invite; *MSPE*, Medical Student Performance Evaluation.

Only 48 (5.4%) of the total applicants had a *meaningful* LTI change, as defined above. One (0.11%) LTI changed from probably no (2) to probably yes (4). Thirty-four LTIs (3.8%) changed from unsure (3) to probably or definitely yes (≥4), and 13 LTIs (1.5%) changed from probably yes or definitely yes (≥4) to unsure, probably no, or definitely no (≤3) (Figure 2, Table 4).

In the 48 applications in which there was *meaningful* change, the most common factor cited for change was MSPE narrative comments in 26 (54.1%) reviews. When there was *no meaningful change* in LTI following MSPE review, the SLOE was the most frequently cited factor for the LTI in 521 (62.8%) of applications reviewed (Table 5).

## DISCUSSION

The MSPE is the only source that provides a comprehensive and comparative assessment of a student’s medical school performance.^13^ Despite the intended purpose, prior work by Shea et al has demonstrated that a significant portion of MSPEs do not clearly state grades and are not transparent regarding whether a student had completed remediation or had adverse actions taken during medical school.^2^ Even though the AAMC clearly outlined the suggested template for MSPE construction across three separate revisions, only 75% of MSPEs followed the proposed guidelines, making it difficult for reviewers to compare students from different medical schools.^9^ Given this variability, the utility of the MSPE in helping to decide which candidates to invite for an interview is likely limited. Our study is the first, to our knowledge, to directly assess the impact of the MSPE on a program’s LTI a residency applicant for an interview.

In addition, it is well recognized that code words used for ranking systems in the MSPE summary statement are largely positive adjectives, even for the lowest performing students. Across medical schools, there is no consistency in what subset of students these positively descriptive terms are referencing.^3^ Our results demonstrate that the MSPE review infrequently results in *meaningful* change in the LTI of an applicant for interview and is strongly suggestive that the utility of the MSPE, as currently constructed, is limited.

Despite repeated guidance from the AAMC for the MSPE to be an *evaluation*, not a *recommendation*, there are incentives for medical schools to present their students in the best light possible.^1^, ^2^ Authors of MSPEs may feel that a student’s inability to match into a residency program reflects poorly on their medical school.^14^ The variability and laudatory nature of the MSPE for even the lowest performers can make it difficult for PDs to use the information provided to effectively screen candidates for interview. Previous literature has gone as far as to suggest that, given the pressure on medical schools to successfully match their students, authors of MSPEs should be an unbiased, knowledgeable group of writers who are not dually conflicted as both student advisors/advocates and evaluators writing the MSPE.^15^

We also know from previous studies that objective factors, such as SLOEs and USMLE scores, have been more influential in a PD’s LTI an applicant for interview. A PD’s reliance upon this data may lie in the fact that these components, unlike the MSPE, are clearly presented and are more useful in quickly comparing applicants across institutions.^8^ Our study corroborates this finding, with the SLOEs driving the decision to extend interviews 62.8% of the time when the MSPE review did not result in any *meaningful* change in LTI. We acknowledge that the true objectivity of the SLOE is still imperfect, as some authors cluster the majority of applicants in the upper tiers of the global assessment ranking and the perceived quality of the narrative is dependent upon the evaluators’ experience and reputation.^16^ Despite these SLOE imperfections, PDs crave succinct, objective, and comparative information when determining the LTI a candidate to interview. Our study reinforces previous work that the SLOE is the primary driver in making these decisions.

In our study, the MSPE review did not frequently result in any *meaningful change* to LTI. In most cases where the MSPE resulted in any change on the Likert scale (n = 110), it was *not a meaningful* change, as determined by the applicant’s likelihood to receive an interview and simply confirmed the decision that had been made *prior* to MSPE review. Interestingly, in both the smaller (n = 48) subset of applicants in which the MSPE did result in *meaningful* change and those where the MSPE resulted in a *non-meaningful* change, the most influential factor was the narrative rotation comments. Perhaps not so coincidentally, this is an area where MSPE authors have been shown to be compliant with the AAMC guidelines, likely reflecting that the information is presented in a format that is easy to interpret and compare between applicants.^3^ Additionally, narrative rotation performance often incorporates aspects of professionalism. Experienced program leaders understand that navigating professionalism issues is among the most challenging of issues to remediate. Given that PDs value high standards of professionalism, adherence to the 2016 AAMC guidelines to include information regarding deficient and exemplary professionalism performance offer an easy opportunity to enhance the utility of the MSPE.

Although the 2020–2021 match cycle included a delayed opening of ERAS with a simultaneous release of the MSPE, traditionally, there has been at least a two-week lag time from the opening of ERAS on September 15 and the release of the MSPE. It is likely that some programs delayed application review during that lag period to wait on the MSPE. Our study results demonstrate that in the majority of applications (94.5%), the MSPE does not result in any *meaningful* change to the LTI, suggesting that PDs could begin application screening and extend interviews prior to MSPE release. The SLOEs are the primary factor influencing the decision to invite applicants, suggesting that the SLOE provides the desired comparative data for applicant reviewers that the MSPE may be lacking.^5^,^17^ It is likely that PDs preferentially appreciate the SLOE, given that it presents information on a student’s medical knowledge, clerkship performance, and professionalism in a succinct and objective format.

As recently published data has shown, applicants have traditionally demonstrated a higher performance on their home rotation when compared to an away rotation.^18^ Traditionally, we have been afforded the opportunity to compare information from an applicant’s home SLOE and at least one away SLOE. Given the restrictions presented by COVID, away rotations were largely prohibited, which limited the ability for applicant reviewers to compare objective data from home versus away rotations. If these restrictions on away rotations continue and only the student’s home SLOE is available to the reviewer, these SLOEs may be perceived as giving a more subjective evaluation of the applicant, as the SLOE authors may want to increase the applicant’s success in matching in their dual roles as evaluators and advisors. If these changes are permanent, perhaps the MSPE, particularly the narrative comments, most closely resembling the narrative comments in the SLOE, will have a bigger impact on applicant LTI in the future.

The MSPE has the potential to provide useful information, but as it currently stands, this letter does not result in *meaningful* change in the LTI for the majority of applicants. Authors of MSPEs undoubtedly spend a significant amount of time constructing this review of a medical student’s performance. Given the time spent and dedication invested by MSPE authors, it would seem prudent that systems be put in place to ensure that the MSPE is truly a reflective evaluation that serves its intended purpose and increases the utility to its readers. If the MSPE were more standardized, objective, inclusive of both positive and negative performance regarding professionalism, easily accessible and discernible, and written by authors who abide by AAMC guidelines, we may obtain the MSPE we have all been yearning for.

## LIMITATIONS

Residency programs have different methods of evaluating applicants and may value different data points when determining the LTI. To assess the impact of the MSPE, reviewers were instructed to view the application while remaining blinded to the MSPE until after they had assigned an LTI score. Reviewers were asked to self-report if they had made an interview decision before looking at the MSPE. Our methods were similar to those outlined in a study evaluating the impact of the standardized video interview and may suffer from similar limitations, most notably a pre-formed notion of the LTI based upon the other elements of the application that may have changed if the MSPE was viewed in a different order.^12^

The LTI and the invite status of an applicant reported in this study were determined from *initial* application review, and thus did not take into account the rare circumstances where an initial invite status was later changed due to specific applicant circumstances, such as an email expressing interest that prompted re-review of the application and ultimate invite or a program moving someone from an on-hold list to fill a last-minute cancellation in the schedule. In these cases, the change in ultimate invite status was not based on the MSPE, but on other extenuating circumstances that changed the application reviewer’s decision. However, given the few instances of these changes occurring, and the MSPE not being the driving factor for these changes, we do not feel that this limitation significantly impacted study results or the validity of the definition of *meaningful* change. Although it could be asserted that the definition of *meaningful* change based on the LTI scale is somewhat subjective, it was shown to accurately represent real-world interview invitation status as shown in Table 4. The applicants ranked as “probably/definitely no” largely ended up ***not*** receiving an interview (99.1% did **not** get an interview) and the applicants ranked “probably/definitely yes” largely ended up receiving an interview (92.7% **did** get an interview). Therefore, a change from “probably no” to “definitely no” and “probably yes” to “definitely yes” was *not a meaningful* change, and further supports our definition of *meaningful* change as outlined above.

We also acknowledge the potential for a Hawthorne effect, as reviewers were not blinded to the purpose of the study during application review. However, there was no effective way for faculty members to be blinded, given that they were asked to determine LTI before and after review of the MSPE, with the only additional data point reviewed in determining the second LTI being the MSPE itself. Finally, although there were three sites in this study, they are all located in a relatively similar geographic location. However, our sample included applicants from 141 distinct institutions, representing all regions of the country.

## CONCLUSION

In a multicenter, prospective, observational study reviewing 877 applications, 94.5% of applications had no *meaningful* change in the likelihood of being invited to interview following MSPE review. For those applications that did have a *meaningful* change, narrative rotation comments were cited as the primary factor. Although we acknowledge that 5% *meaningful* change is not completely insignificant, the extensive time involved in detailed MSPE review overall results in infrequent change in an applicant’s LTI. Perhaps a renewed call for MSPE authors to adhere to the guidelines, with an emphasis on providing consistently organized and objective content, would result in a higher frequency of *meaningful* change in LTI, justifying the time spent by program leaders in reviewing this document. In conclusion, although the MSPE has the potential to provide comparative and objective information regarding medical school performance, review of the MSPE in its current construct infrequently results in *meaningful* change in the likelihood to invite an applicant for interview.

## Supplementary Information





## Figures and Tables

**Table 1:** Demographic characteristics of the three participating program sites and application reviewers at the respective sites.

	Site 1	Site 2	Site 3
Program length	3	3	3
Program class size	12	10	10
Setting	Community/university-affiliated, urban	University, urban	University, rural
Total applications received	1,191	1,071	643
Applications reviewed n, (% of total)	244 (20.4%)	290 (27.1%)	343 (53.3%)
Reviewers	Program Director, Associate Program Director, Chief resident	Program Director, Assistant Program Director	Program Director, Associate Program Director
Years of experience of each reviewer	PD-20 years APD-10 years Chief resident-1 year, supervised by PD and APD	PD-13 years APD-4 years	PD-9 years APD-8 years

*PD*, program directors; *APD*, assistant/associate program directors.

**Table 2:** Demographic characteristics of the applicants.

Total number of unique applicants reviewed	757
Age (range in years)	23–48
Mean Age, SD	27.8 ±3.2
Gender, n (%)	
Male	487 (64.4%)
Female	269 (35.5%)
Region, n (%)	
Northeast	182 (24.0%)
Southeast	234 (30.9%)
Midwest	201 (26.5%)
West	135 (17.8%)
International	4 (0.52%)
Medical school type, n (%)	
Public	509 (67.2%)
Private	179 (23.6%)
Osteopathic	64 (8.4%)
International	4 (0.52%)
Standardized examination scores, range (mean SD +/−)	
USMLE Step 1	192–265 (231 ± 15)
USMLE Step 2 CK	210–279 (244 ± 14)
COMLEX Level 1	451–730 (601 ± 68)
COMLEX Level 2	423–887 (625± 96)

*USMLE*, United States Medical Licensing Examination; *CK*, clinical knowledge; *COMLEX*, Comprehensive Osteopathic Medical Licensing Examination; *SD*, standard deviation.

**Table 3:** Descriptive statistics correlating final Likert scale “likelihood to invite” ratings with “real-world” applicant interview status.*

Final LTI after MSPE review	Received interview invitation (n, % of LTI category)	No interview invitation received (n, % of LTI category)
Definitely no	0 (0%)	106 (100%)
Probably no	3 (1.5%)	197 (98.5%)
Still unsure	27 (20.0%)	108 (80.0%)
Probably yes	217 (89.7%)	25 (10.3%)
Definitely yes	187 (96.4%)	7 (3.6%)
Total	434	443

* Note that interviews that were extended after the November 1 conclusion of this study were considered to be a “no invite received” for the purpose of this analysis.

*LTI*, likelihood to invite; *MSPE*, Medical Student Performance Evaluation.

**Table 4:** Effect of Medical Student Performance Evaluation on likelihood to invite (LTI) and characteristics of LTI change.

	N (%)	95% CI	*P*
MSPE review resulted in no meaningful change on LTI	829 (94.5%)	92.8–95.8	<0.001
MSPE review resulted in meaningful change on LTI overall	48 (5.5%)	4.1–7.2	
LTI changed from definitely/probably no or unsure to definitely/probably yes	35 (3.9%)	2.8–5.4	
LTI changed from definitely/probably yes to unsure or from definitely/probably yes or unsure to definitely/probably no	13 (1.5%)	0.8–2.5	

*LTI*, likelihood to invite; *MSPE*, Medical Student Performance Evaluation; *CI*, confidence interval.

**Table 5:** Primary factor in decision to invite if there was *no meaningful* change in LTI after MSPE review and primary factor obtained from MSPE if *meaningful* LTI changed after MSPE review.

Primary factor in decision to invite if *no meaningful* change (no change at all + insignificant-*not meaningful* change) in LTI after MSPE review (total n=829)	N (%)
Non-MSPE Factors	
SLOE global assessment	521 (62.8%)
USMLE performance	49 (6.0%)
Prior knowledge of applicant from rotation	24 (2.9%)
Aspects of CV (research, awards)	20 (2.4%)
Personal statement	15 (1.8%)
Other	90 (10.9%)
MSPE Factors	
Additional character information	8 (1.0%)
Class ranking	23 (2.8%)
Delay in completion of training	1 (0.1%)
Narrative rotation comments	47 (5.7%)
Other	11 (1.3%)
Perception of professionalism	5 (0.6%)
Report of remediation	15 (1.8%)
Primary factor obtained from MSPE if MSPE review resulted in *meaningful* change (total n = 48)	
Narrative rotation comments	26 (54.2%)
Class ranking	11 (23.0%)
Report of remediation or probation	3 (6.3%)
Additional character information (mission trips, background, volunteerism)	3 (6.3%)
Perception of professionalism	4 (8.3%)
Other	1 (2.1%)

*LTI*, likelihood to invite; *MSPE*, Medical Student Performance Evaluation; *SLOE*, Standard Letter of Evaluation; *USMLE*, US Medical Licensing Examination; *CV*, curriculum vitae.
