# Supplementary material for: Does the Medical Student Performance Evaluation Change the Decision to Invite Residency Applicants?
Source: West J Emerg Med. 2021 Aug 21;22(5):1102–9. doi: 10.5811/westjem.2021.4.52374 (PMC8463047; doi:10.5811/westjem.2021.4.52374)
Supplement: Supplementary file 1 [file wjem-22-1102-s001.docx]

MSPE decision to invite data collection

Start of Block: Default Question Block

Q1 Program reviewing this application

▼ West Virginia University (4) ... Medical University of South Carolina (7)

Q2 AAMC ID of applicant

________________________________________________________________

Q9 Have you extended an official interview invitation to this applicant prior to reviewing the MSPE? If yes, click yes, the portal will allow you to skip the remainder of the questions and then please hit submit.

- Yes (1)

Display This Question:

If Have you extended an official interview invitation to this applicant prior to reviewing the MSPE?... != Yes

Q3 Did you previously know this applicant? (i.e. rotator at your institution or medical student at your institution)

- Yes (1)
- No (2)

Display This Question:

If Have you extended an official interview invitation to this applicant prior to reviewing the MSPE?... != Yes

Q4 Review the application as you normally would without reviewing the MSPE.  Are you planning to invite this applicant for an interview at your program?

- Definitely no (1)
- Probably no (2)
- Unsure on initial review (3)
- Probably yes (4)
- Definitely yes (5)

Display This Question:

If Have you extended an official interview invitation to this applicant prior to reviewing the MSPE?... != Yes

Q5 After determining how likely you are to invite the applicant without MSPE review, now review the MSPE.  After MSPE review, are you planning to invite this applicant for an interview at your program?

- Definitely no (1)
- Probably no (2)
- Still unsure (3)
- Probably yes (4)
- Definitely yes (5)

Display This Question:

If Have you extended an official interview invitation to this applicant prior to reviewing the MSPE?... != Yes

Q6 If your answer to how likely you are to invite the candidate changed after review of the MSPE, what information from the MSPE made you change your mind?

- Narrative rotation comments (4)
- Class ranking (5)
- Report of remediation or probation (6)
- Delay in completion of training (7)
- Perception of professionalism (9)
- Additional character information (background, volunteerism, medical mission trips) (11)
- Other (specify in comments) (10) ________________________________________________

Display This Question:

If Have you extended an official interview invitation to this applicant prior to reviewing the MSPE?... != Yes

Q7 For those applicants that the MSPE did not change your mind, what was the primary data point in your decision to invite or not invite?

- SLOE global assessment/position on rank list (1)
- Personal statement (2)
- Prior knowledge of applicant--i.e. completed rotation at your site, medical student at your site (3)
- CV--activities, prior awards, research (4)
- USMLE performance (5)
- Other (6) ________________________________________________

End of Block: Default Question Block
