# Supplementary material for: Does the Medical Student Performance Evaluation Change the Decision to Invite Residency Applicants?
Source: West J Emerg Med. 2021 Aug 21;22(5):1102–9. doi: 10.5811/westjem.2021.4.52374 (PMC8463047; doi:10.5811/westjem.2021.4.52374)
Supplement: Supplementary file 2 [file wjem-22-1102-s002.docx]

Appendix B: Geographical demographic information of institutions attended by applicants in the study population.

Running total by state

Southeast

Alabama

1. Alabama School of Osteopathic Medicine—3
2. University of Alabama at Birmingham—7
3. University of South Alabama—3

**Alabama total: 13**

**Osteopathic: 3**

**Public: 10**

Georgia

1. Emory---1
2. Medical College of Georgia at Augusta University—10
3. Mercer—4
4. Morehouse—2

**Georgia total: 17**

**Osteopathic: 0**

**Public: 10**

**Private: 7**

North Carolina

1. Campbell University—3
2. Duke-1
3. East Carolina University—6
4. University of North Carolina—7
5. Wake Forest—4

**North Carolina total: 21**

**Osteopathic: 3**

**Public: 13**

**Private: 5**

Puerto Rico

1. Ponce—1
2. San Juan Battista—1
3. University of Puerto Rico—2

**Puerto Rico total: 4**

**Private: 2**

**Public: 2**

South Carolina

1. Medical University of South Carolina—6
2. University of South Carolinia—18

**South Carolina total: 24**

**Public: 24**

Florida

1. Florida Atlantic—6
2. Florida International—8
3. Florida State University—7
4. Nova Southeastern College of Osteopathic Medicine—1
5. University of Central Florida—5
6. University of Florida-3
7. University of South Florida—8
8. University of Miami—7

**Florida Total: 45**

**Osteopathic: 1**

**Public: 37**

**Private: 7**

Louisiana

1. Louisiana State University—5
2. Tulane—2

**Louisiana total: 7**

**Public: 5**

**Private: 2**

Arkansas

1. University of Arkansas for Medical Sciences—5

**Arkansas total: 5**

**Public: 5**

Tennessee

1. East Tennnessee State University—4
2. Meharry—2
3. University of Tennessee.—8
4. Vanderbilt University—3

**Tennesssee total: 17**

**Public: 12**

**Private: 5**

Kentucky

1. University of Kentucky—9
2. University of Louisville—12

**Kentucky total: 21**

**Public: 21**

West Virginia

1. Marshall University—6
2. West Virginia University—11
3. West Virginia School of Osteopathic Medicine—1

**West Virginia total: 18**

**Osteopathic: 1**

**Public: 17**

Virginia

1. Eastern Virginia Medical School -11
2. Edward Via Osteopathic—6
3. Virginia Commonwealth University—10
4. Liberty University College of Osteopathic Medicine—1
5. University of Virginia: 3
6. Virginia Tech Carilion—4

**Virginia total: 35**

**Osteopathic: 7**

**Public: 28**

Mississippi

1. University of Mississippi—6
2. Wm. Carey College of Osteopathic Medicine—1

**Mississippi total: 7**

**Public: 6**

**Osteo: 1**

**Southeast total: 234**

**Northeast**

Maine

1. University of New England School of Osteopathic Medicine—1

**Maine total: 1**

**Osteopathic: 1**

New Hampshire

1. Dartmouth—2

**NH total: 2**

**Private: 2**

Vermont

1. University of Vermont—10

**Vermont total: 10**

**Public: 10**

Massachusetts

1. Boston University—3
2. Tufts—4
3. University of Massachussetts-4

**Massachusetts total: 11**

**Private: 7**

**Public: 4**

Connecticut

1. Quinnipiac—3

**Connecticut total: 3**

**Private: 3**

New York

1. Albany Medical College—9
2. Hofstra University—3
3. Icahn Mt. Sinai—2
4. SUNY Buffalo—9
5. NY Tech College of Osteopathic Medicine—1
6. NY Med Coll—1
7. SUNY Downstate—1
8. SUNY Upstate—7
9. Touro NY College of Osteopathic Medicine—1
10. University of Rochester—1

**New York total: 35**

**Osteopathic: 2**

**Public: 17**

**Private: 16**

New Jersey

1. Cooper Rowan—8
2. Rowan Osteopathic—3
3. Rutgers—2

**New Jersey total: 13**

**Osteopathic: 3**

**Public: 10**

Pennsylvania

1. Drexel—4
2. Geisinger—8
3. Jefferson—15
4. Lake Erie College of Osteopathic Medicine—19
5. Temple—4
6. Penn State—6
7. University of Pennsylvania-3
8. Philadelphia College of Osteopathic Medicine—13
9. University of Pittsburgh—7

**Pennsylvania total: 79**

**Private: 30**

**Public: 17**

**Osteopathic: 32**

District of Columbia

1. George Washington—6
2. Georgetown—8
3. Howard—5

**District of Columbia total: 19**

**Private: 19**

Maryland

1. Johns Hopkins—1
2. Uniformed Services University of Health Sciences—3
3. University of Maryland—5

**Maryland total: 9**

**Private: 1**

**Public: 8**

**Northeast total: 182**

**Midwest**

Ohio

1. Case Western—9
2. Northeastern Ohio Universities College of Medicine-11
3. Ohio State—5
4. Ohio University Heritage College of Osteopathic Medicine—1
5. University of Toledo—9
6. University of Cincinnati —10
7. Wright State—9

**Ohio total: 54**

**Osteopathic: 1**

**Public: 44**

**Private: 9**

Indiana

1. Indiana University—14

**Indiana total: 14**

**Public: 14**

Illinois

1. Midwestern University/Chicago College of Osteopathic Medicine—1
2. Rosalind Franklin University/Chicago College of Medicine—11
3. Loyola—4
4. Rush—12
5. Southern Illinois University— 5
6. University of Illinois—8 (all campuses)

**Illinois total: 41**

**Osteopathic: 1**

**Public: 13**

**Private: 27**

Wisconsin

96. Medical College of Wisconsin—11

97. University of Wisconsin —11

**Wisconsin total: 22**

**Public: 11**

**Private: 11**

Michigan

98. Central Michigan—4

99. Michigan State—4

100. Oakland University William Beaumont-4

101. University of. Michigan—3

102. Wayne State—7

103. Western Michigan—2

**Michigan total: 24**

**Public: 16**

**Private: 8**

Minnesota

104. University of Minnesota—4

**Minnesota total: 4**

**Public: 4**

Iowa

105. University of Iowa—7

**Iowa total: 7**

**Public: 7**

Missouri

106. Kansas City College of Osteopathic—4

107. Saint Louis University—4

108. University of Missouri—8

**Missouri total: 16**

**Osteopathic: 4**

**Public: 8**

**Private: 4**

Kansas

109. University of Kansas—12

**Kansas total: 12**

**Public: 12**

Nebraska

110. Creighton—3

**Nebraska total: 3**

**Private: 3**

South Dakota

111. University of South Dakota —4

**SD total: 4**

**Public: 4**

**Midwest total: 201**

**Southwest**

Texas

112. Baylor College of Medicine—4

113. UT Houston—8

114. Texas A&M—11

115. Texas Tech—9

116. UTSA—7

117. Other University of Texas branches-19

**Texas total: 58**

**Private: 4**

**Public: 54**

Colorado

118. University of Colorado—8

**Colorado total: 8**

**Public: 8**

Utah

119. University of Utah 7

**Utah total: 7**

**Public: 7**

New Mexico

120. University of New Mexico—6

**New Mexico total: 6**

**Public: 6**

Arizona

121. Arizona College of Osteopathic Medicine of Midwestern University—1

122. University of Arizona—6

**Arizona total: 7**

**Osteopathic: 1**

**Public: 6**

Nevada

123. University Nevada at Reno—3

**Nevada total: 3**

**Public: 3**

California

124. University of Southern California—2

125. California Northstate—1

126. Loma Linda—4

127. UC Davis—2

128. UC Irvine—2

129. UCSF—5

130.Western University/College of Osteopathic Medicine of the Pacific-2

**California total: 18**

**Osteopathic: 2**

**Private: 7**

**Public: 9**

Hawaii

131. University of Hawaii—4

**Hawaii total: 4**

**Public: 4**

**Northwest**

Oregon

132. Oregon Health Sciences University—4

133. Pacific Northwest University College of Osteopathic Medicine—1

**Oregon total: 5**

**Osteopathic: 1**

**Public: 4**

Washington

134. University of Washington 19

**Wash total: 19**

**Public: 19**

**West total: 135**

**International**

135. Ross—1

136.St. George London—2

137. St. Kitts-1

**International: 4**

**141 total schools with 4 total UT branches**

**One application missing school attended information
